# Attitudes of anesthetists towards an anesthesia-led nurse practitioner model for low-risk colonoscopy procedures: a cross-sectional survey

**DOI:** 10.1186/s12960-020-0458-1

**Published:** 2020-03-17

**Authors:** L. Weinberg, H. Grover, D. Cowie, E. Langley, M. Heland, D. A. Story

**Affiliations:** 1 Department of Anesthesia, Austin Hospital, 145 Studley Road, Heidelberg, Victoria 3084 Australia; 2 Department of Surgery, The University of Melbourne, Melbourne, Victoria Australia; 3 Department of Anesthesia Perioperative and Pain Medicine Unit, The University of Melbourne, Melbourne, Victoria Australia; 4 Centre for Integrated Critical Care, The University of Melbourne, Melbourne, Victoria Australia

**Keywords:** Anesthetist, Nurse, Sedation, Colonoscopy, Propofol

## Abstract

**Background:**

The mounting pressure on the Australian healthcare system is driving a continual exploration of areas to improve patient care and access and to maximize utilization of our workforce. We hypothesized that there would be support by anesthetists employed at our hospital for the design, development, and potential implementation of an anesthesia-led nurse practitioner (NP) model for low-risk colonoscopy patients.

**Methods:**

We conducted a cross-sectional, mixed methods study to ascertain the attitudes and acceptability of anesthetists towards a proposed anesthesia-led NP model for low-risk colonoscopy patients. An online survey using commercial software and theoretical questions pertaining to participants’ attitudes towards an anesthesia-led NP model was e-mailed to consultant anesthetists. Participants were also invited to participate in a voluntary 20-min face-to-face interview.

**Results:**

A total of 60 survey responses were received from a pool of 100 anesthetists (response rate = 60%, accounting for 8.04% margin of error). Despite the theoretical benefits of improved patient access to colonoscopy services, most anesthetists were not willing to participate in the supervision and training of NPs. The predominant themes underlying their lack of support for the program were a perception that patient safety would be compromised compared to the current model of anesthesia-led care, the model does not meet the Australian and New Zealand College of Anesthetists guidelines for procedural sedation and analgesia, and the program may be a public liability prone to litigation in the event of an adverse outcome. Concerns about consumer acceptance and cost-effectiveness were also raised. Finally, participants thought the model should be pilot tested to better understand consumer attitudes, logistical feasibility, patient and proceduralist attitudes, clinical governance, and, importantly, patient safety.

**Conclusions:**

Most anesthetists working in a single-center university hospital did not support an anesthesia-led NP model for low-risk colonoscopy patients. Patient safety, violations of the current Australian and New Zealand College of Anesthetists guidelines on procedural sedation, and logistical feasibility were significant barriers to the acceptance of the model.

**Trial registration:**

Australian and New Zealand Clinical Trials Registry, 12619001036101

## Introduction

With an ever-increasing demand for surgical and medical diagnostic and/or interventional procedures, there is a mounting pressure on hospitals worldwide to maximize utilization of their healthcare workforce through different service provider models [1–4]. Aligned with the recommendations of the United States Preventive Services Task Force regarding screening for bowel cancer [5], the Australian National Bowel Cancer Screening Program’s strategic initiative invites all Australians between 50 and 74 years of age to screen for bowel cancer every 2 years [6]. Worldwide, the future demand for screening and surveillance for endoscopy procedures is therefore expected to escalate as international bowel cancer screening programs continue to be fully implemented. In an Australian context, service models for colonoscopy may not meet demands in the current public health framework [6].

Internationally, healthcare systems and professional practice organizations have responded to this type of demand through the development and implementation of nurse practitioner (NP) educational programs. Such programs have clear definitions regarding clinical nurse competencies, responsibilities, and practice guidelines [7–10]. In Australia, mandatory requirements for an NP include registration with the Nurses and Midwives Board of Australia as a registered nurse, experience in a clinical specialty, and education at the master’s level [11].

Currently, NPs in Australia work in the following specialty areas: emergency medicine, aged care, drug and alcohol, surgical, rural and remote, women’s health, mental health, midwifery, and pediatrics. Their role differs from “traditional” models of nursing care by additional responsibilities and competencies that include the performance of advanced health assessments, initiation, and interpretation of diagnostic investigations such as pathology and diagnostic imaging, diagnosing health problems, designing, implementing and monitoring of therapeutic regimens in collaboration with patients, families/carers and other health professionals, prescribing medications, and the initiation and receival of appropriate referrals to and from other health professionals. The expansion of the NP roles has resulted in an increase in nursing expertise, high-quality patient outcomes, and an improved multidisciplinary approach to healthcare [12]. Importantly, there is no existing evidence of the acceptability of these advanced nursing roles by Australian physicians.

While there is confusion in the international literature regarding the nomenclature of NPs in anesthesia, as well as a lack of standardizations regarding specific training and competencies, NP roles are now well-accepted models of care in numerous continents across the globe. In the United States of America, NPs in anesthesia are commonly acknowledged as certified registered nurse anesthetists (CRNAs), whereas in Canada, France, and the United Kingdom, NPs in anesthesia are referred to as anesthesia assistants, infirmier anesthesiste diplome d’etat, and physicians’ assistant in anesthesia, respectively [13].

Presently in Australia, there is no formal recognition of the role of NP in anesthetics, and the administration of anesthesia medications, such as propofol, by registered nurses is constrained by significant regulatory considerations and guidelines, in addition to intense pressure from various anesthesia craft groups [14]. Advanced nurse practitioner roles in anesthesia are therefore very limited in Australia and confined to very few hospitals, each with their own local governance and training programs. In these specific settings, the NP provides anesthesia care for low-risk patients, most of whom are undergoing endoscopic procedures such as colonoscopy. In such settings, it has been reported that nurse-administered procedural sedation is safe and effective, findings supported by numerous international studies exploring the safety of non-anesthetists using propofol for sedation for colonoscopy patients [8, 9, 15–18]. A recent independent Cochrane review examining the safety of anesthesia when administered by PNs versus physicians concluded that given the low intrinsic rate of complications relating directly to anesthesia together with the complexity of perioperative care, no definitive statement can be made concerning the possible superiority of one anesthesia care provider over another [19].

**Fig. 1:**
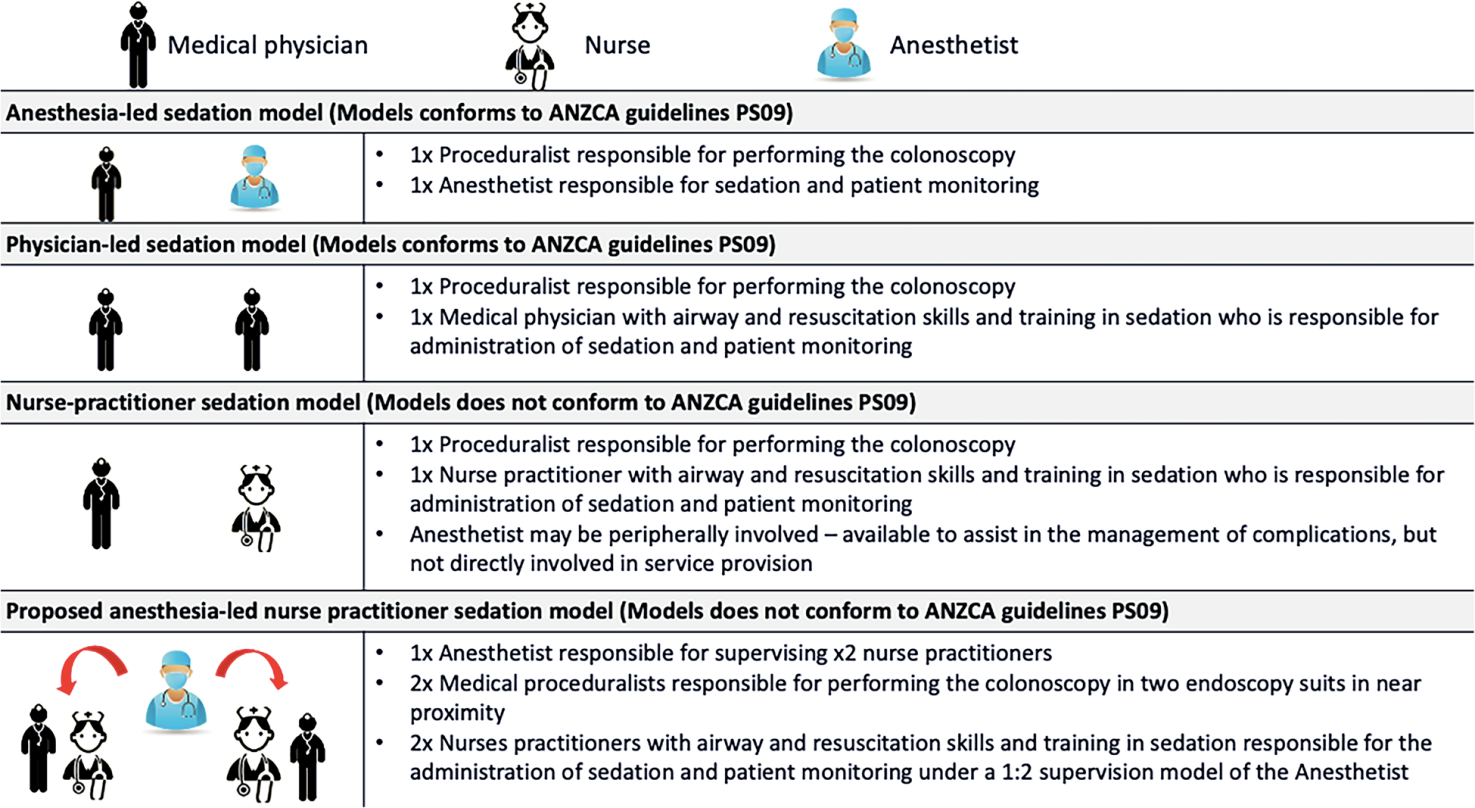
Models of sedation for endoscopy

Given that the future demand for screening and surveillance for endoscopic procedures is expected to escalate in Australia, this prompted a reconsideration of current models of service delivery for patients undergoing colonoscopy at our university teaching hospital. Accordingly, a working group in our hospital formed to explore implementation strategies to further increase access for patients undergoing elective colonoscopy. The collaborative working group consisted of a senior nurse, two anesthetists, a surgeon and a gastroenterologist, and a senior hospital executive. During this 4-month consultation process, four models of sedation were outlined (Fig. 1), which provided a practical framework in the planning, execution, and potential implementation of any change to future practice. One such proposed strategy from the working group for increasing service delivery to patients requiring elective colonoscopy was the development of an anesthesia-led NP program.

However, after an extensive review of the literature, consultations, and site visits to other hospitals in Australia that has implemented similar programs, the working group considered that the proposed program could be contentious at our hospital for the following reasons. First, under a conventional “anesthetist-managed” sedation model with propofol, significant complications were reported in a large study investigating patients undergoing elective and emergency gastrointestinal endoscopy in a university teaching hospital similar to our own [20]. Second, the current position of the Australian and New Zealand College of Anesthetist (ANZCA) is that the provision of anesthesia is a “medical” role [21]. Third, the development of an anesthesia-led nurse sedation model using propofol currently failed to comply with ANZCA guidelines on sedation for diagnostic and interventional medical and surgical procedures [22]. To further explore the barriers and enablers that could influence the implementation of such a program, we conducted a cross-sectional, mixed methods study to ascertain the attitudes and acceptability of anesthetists working in our hospital towards a proposed anesthesia-led NP model for low-risk colonoscopy patients. We hypothesized that there would be in-principle support by anesthetists employed at our hospital for the design, development, and potential implementation of an anesthesia-led NP model.

## Methods

### Study population

After obtaining Austin Health Human Research Ethics Committee approval (LNR/17/Austin/224, approved 2 June 2017), we conducted a prospective mixed methods survey of practicing fellow anesthetists at Austin Health, a university teaching hospital. Austin Health provides tertiary-level services for adult patients across all major specialties, including hepatobiliary surgery and liver transplantation, cardiothoracic surgery, spinal surgery, and major upper and lower gastrointestinal surgery. The hospital undertakes approximately 8000 endoscopic procedures annually, across two hospital campuses. Participants were registered with ANZCA and working in our department as full time or sessional consultant anesthetists. Anesthesia registrars and trainees were excluded, as were fellows who were retired or appointed in honorary roles. The study was registered with the Australian and New Zealand Clinical Trials Registry (Trial number: ACTRN12619001036101).

### Survey distribution

After an extensive literature review about the safety of the anesthesia-led NP program and site visits to two Australian hospitals that provided an NP service for colonoscopy patients, we designed an online survey using a commercial software (SurveyMonkey Inc., San Mateo, CA, USA), which was then pilot tested on five randomly selected anesthetists from within our hospital. The final questionnaire consisted of six theoretical questions that pertained to participants’ attitudes towards an anesthesia-led AP model, along with an invitation to participate in a voluntary 20-min face-to-face interview.

An invitation e-mail that included a summary and objectives of the study, information about consent, and a link to the online survey was sent directly to the consultant anesthetists in our hospital. A copy of the invitation e-mail is presented in Appendix 1. To avoid acquiescence and social bias, the e-mail was sent by an independent project manager, who was not known to any of the anesthetists. The e-mail was sent to 100 consultant anesthetists, excluding the five anesthetists who had participated in the pilot testing of the survey. The survey was open from 5 to 19 June 2017. During this time, participants were able to access and complete the survey at their convenience. They could only complete the survey once, thereby negating any risk of response duplication. No reminder e-mails were sent nor was compensation offered for participation in the study. All responses were completely anonymous, and no Internet Protocol (IP) addresses were collected.

### Survey scenario

The online survey provided participants with a proposed theoretical scenario stating that a training curriculum would be designed and supervised by the department of anesthesia in conjunction with an affiliated university to train NPs in procedural sedation. A consultant anesthetist would be responsible for providing 1:2 supervision for trained registered nurse specialists providing procedural sedation (including propofol) for low-anesthetic risk patients undergoing colonoscopy procedures. Low risk was defined as adult patients (aged >18 years), American Society of Anesthesiology classes I and II, with a body mass index of < 35 kg/m^2^, no previous history of complications from anesthesia, and no known or predicted difficult airway. Members from the department of anesthesia were invited to comment on the acceptability of a proposed model of anesthesia-led nurse sedation in lower gastrointestinal endoscopy.

### Survey statements

Through the online survey, members from the department of anesthesia were invited to comment on the acceptability of a proposed model of the anesthesia-led NP model in lower gastrointestinal endoscopy. Participants were provided with the six following statements:

- I am familiar with the Australian and New Zealand College of Anesthetists document PS09—Guidelines on Sedation and/or Analgesia for Diagnostic and Interventional Medical, Dental or Surgical Procedures.
- This model of care could be comparable in safety compared to an anesthetist only model, which is the current standard of care.
- I would be prepared to act as a supervisor for two appropriately trained nurses administering procedural sedation, including propofol.
- I would be prepared to participate in training appropriate nursing candidates for an anesthesia-led nurse sedation role.
- An anesthesia-led nurse sedation model could deliver procedural sedation in its entirety—including patient preparation and assessment and procedural and post-procedural requirements.
- I support the exploration of alternative service models in procedural sedation in an effort to meet demand in the current health economic climate.

A 5-point Likert scale (*strongly agree* to *strongly disagree*) was used to determine the attitudes of anesthetists towards the anesthesia-led NP model. A free-text box was included at the end of the survey, allowing the respondents to comment on any aspects of the proposed model. Finally, all participants were asked to volunteer for further face-to-face interviews and, if interested, were asked to leave their preferred contact details in a dedicated space within the survey.

### Conduct of interviews

Participants who volunteered for the face-to-face survey were contacted by one of the principal investigators (DC), who was responsible for organizing interview dates and times. Interview times were flexible across the study period to accommodate the participants’ clinical demands. Informed consent for interviews was obtained, and all interviews were audio-recorded on dedicated for purpose recorder and then transcribed following the interview. All audio files were deleted immediately following the transcription. The detailed interview script was approved by the Research Ethics Committee and is summarized in Table 1.

**Table 1:** Interview script

We are testing a hypothetical model to determine the acceptability of an anesthesia-led nurse sedation model. The proposed model is that a consultant anesthetist is responsible for providing 1:2 supervision for trained registered nurses providing procedural sedation (including propofol) for low-anesthetic risk patients undergoing colonoscopy.	
You recently completed an online survey that asked explored your levels of agreement or disagreement with six statements.	
**Question 1.** An anesthesia-led nurse sedation model could be comparable in safety compared to an anesthetist only model, which is the current standard of care. What would we need to ensure that any alternative model is designed with safety at the forefront? What elements are required for safe non-anesthetist led procedural sedation?	
**Question 2.** Where does this service model fit with PS09? Do you think the PS09 is an appropriate guideline for procedural sedation?	
**Question 3.** What level of supervision do you believe would be needed to ensure the safety and quality of nurse procedural sedation for elective colonoscopy patients?	
**Question 4.** Refers to ANZCA levels of supervision. What training do you believe non-anesthetist clinicians need to be adequately trained in procedural sedation? i.e., masters level, in-house training, training associated with a university, hands-on/practical training, and simulation training.	
**Question 5.** An anesthesia-led nurse sedation model could deliver procedural sedation in its entirety, including patient preparation and assessment and procedural and post-procedural requirements. Could an appropriately trained registered nurse specialist complete all elements of procedural sedation required?	
**Question 6.** Do you support the exploration of alternative service models in procedural sedation in an effort to meet demand in the current health economic climate? Have you considered alternative service models? Please comment on any benefits, challenges, or risks you foresee for an anesthesia-led nurse sedation model at our hospital.	

To avoid any interviewer or interviewee bias, all interviews were conducted by a skilled project officer (EM) external to both the department of anesthesia and the hospital executive. The project officer was not known to any of the anesthetists and was trained and skilled in interview techniques. The interviews were standardized by an a priori interview script that had also been pilot tested on the same five anesthetists who had participated in the original pilot survey. Interviews were conducted either in a private meeting room located within the department of anesthesia or in the anesthetist’s private office at the request of the participants.

### Data analysis

Data for age distribution, gender, and years of practice as a consultant anesthetist were obtained. The responses received from the pilot test were excluded. Statistical analysis was performed using commercial statistical software with a *P* value of 0.05 to indicate statistical significance. Data is presented as frequencies and percentage values. No survey weighting adjustment was conducted, due to the unavailability of the appropriate auxiliary variables and a lack of detailed population reference data. As this was an exploratory approach to qualitative data analysis, the specific analytic themes were not predetermined but were derived from the data. NVivo v.12 (QSR International Pty Ltd, Burlington, USA) was employed to extract the main themes from the free-text and interview responses. The identified themes were summarized and used to guide decision-making for the future development of service models.

## Results

A total of 60 survey responses were received from a pool of 100 consultant anesthetists (response rate = 60%, accounting for 8.04% margin of error). All participants (100%) answered every question on the online survey. Of the participants, 40 (67%) were male, and 20 (33%) were female. The age distribution of participants was 30 to 39 years (32%), 40 to 49 years (43%), 50 to 59 years (18%), and 60 years or older (5%). Thirty-nine participants (65%) completed the free-text box section of the survey, and 24 participants (40%) accepted the invitation for a follow-up, confidential, face-to-face interview.

### Online survey responses

**Fig. 2:**
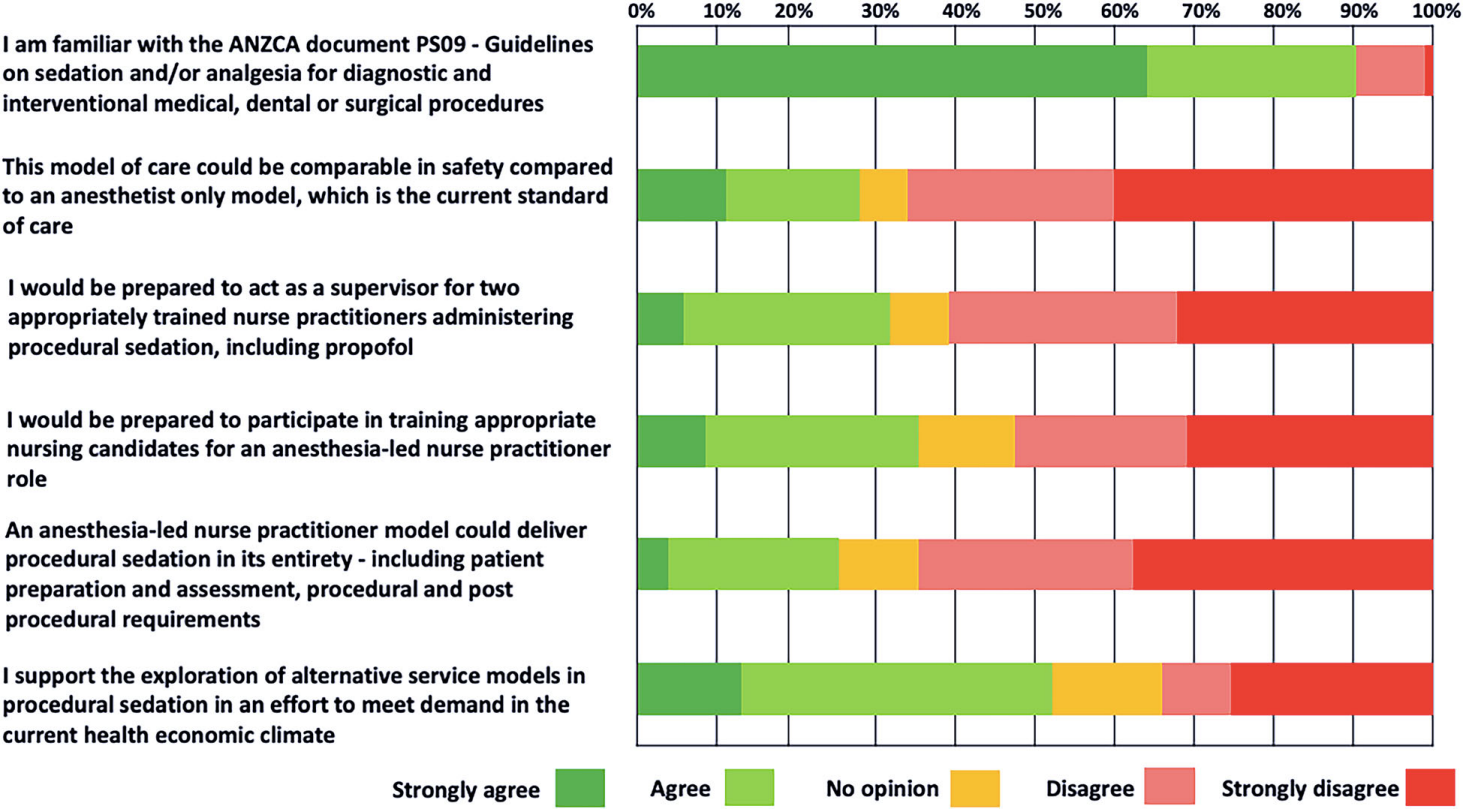
Summary of the online survey results

The survey statements using a 5-point Likert scale exploring attitudes of anesthetists towards an anesthesia-led NP model for low-risk patients undergoing is presented in Fig. 2. Despite the theoretical benefits of improved patient access to colonoscopy services presented in the hypothetical survey scenario, most anesthetists considered that the proposed anesthesia-led NP model would not be comparable to the current anesthetists, i.e., physician-led model. Further, most anesthetists were not willing to participate in the supervision and training of NPs. The predominant themes underlying their lack of support for the program were a perception that patient safety would be compromised compared to the current model of anesthesia-led care, the model did not meet the ANZCA guidelines for procedural sedation and analgesia, and the program may be a public liability prone to litigation in the event of an adverse outcome. Concerns about consumer acceptance and cost-effectiveness were raised. Finally, participants thought the model should be pilot tested to better understand consumer attitudes, logistical feasibility, patient and proceduralist attitudes, clinical governance, and, importantly, patient safety.

### Free-text responses

After completing the online survey, 24 participants entered further comments as free text. These responses were categorized into three key themes, namely safety considerations, economic considerations, and clinical governance considerations. Participants felt that performing outside of the ANZCA guidelines would leave the department of anesthesia open to scrutiny from other hospitals and anesthesia departments. Some participants suggested that nurse sedation outside of the ANZCA guidelines would be a public liability that would open the door to litigation in the event of adverse outcomes. Free-text responses exploring attitudes of anesthetists towards the proposed anesthesia-led NP model for low-risk elective colonoscopy patients are summarized in Table 2.

**Table 2:** Free-text responses exploring attitudes of anesthetist towards a proposed anesthesia-led advanced nurse practitioner model for low-risk elective colonoscopy patients

Safety	Health economics	Clinical governance
“The major concern is the ability of non-anesthetists to manage the airway in the event of laryngospasm or apnea.” “Endoscopy is sometimes associated with significant sedation-related complications e.g. hypoxia, hypotension, bradycardia. A medically trained expert should be responsible for their management.” “If the patients are high-risk, a non-anesthetist model for sedation will not be as safe as an anesthetist-led model” “Complications during sedation for endoscopy can happen quickly. The patient population for endoscopy often involves sicker, older and more obese patient with significant comorbidities. These cases are challenging for highly skilled anesthetists.” “As a community, if we wish to prioritize endoscopy within the constraint of limited resources, compromising safety is not acceptable.” “The model could only work if all patients are low risk.” “I think we should appreciate that colonoscopy by anesthetists is usually a "general anesthesia with propofol”. “Sedation may appear easy, but this is only because anesthetists are highly skilled and trained. It’s important to recognize sedation related complications are common especially if propofol is being used.” “Propofol should not be used by anybody who is not proficient in airway management” “The reason why anesthesia is safe in Australia is because anesthesia provision for all patients is based on a one-doctor to one-patient relationship”. “Who will take ultimate responsibility for any sedation related complications on these lists?”	“The patient must come first not the dollar.” “A cost analysis of this model must be undertaken evaluating the proposed program - nurse training time, development of a structured program, costs of nurse specialists, costs of the anesthetists who teach on the course, content and the training. The model then needs to be tested clinically as part of an ethically approved quality improvement programme.” “There are many factors that impact on endoscopy waiting times and efficiency of endoscopy lists. These include, but are not limited, to waiting for equipment turnover, proceduralist skill, whether the lists are training or teaching lists, patient complexity, and patient pathology. The anesthetist is often not the rate limiting step in efficiency.” “We should not consider lowering the current standard of excellent care if we are not certain this proposed model will be safe. I do not think that the model is economically more efficient.” “I can see why a non-anesthetist sedation model is appealing to management. It appears to be cheaper, but have any economic analyses taken into account sedation-related complications and potential litigation as a result of sedation without an anesthetist? What will patients think?”	“This program would not comply with current ANZCA guidelines for procedural sedation.” “Any new model of sedation must comply with ANZCA guidelines on procedural sedation.” “Any proposed changed to an anesthetist-led service for colonoscopy needs to be discussed with the patients and endoscopists. We need to explore their views as well.” “Any new model should not compromise the training of our anesthesia residents and registrars.” “The use of anesthesia registrars with 2:1 supervision would be an alternative and acceptable model.” “I would be prepared to act as a supervisor for two anesthetic registrars administering procedural sedation, including propofol.” “There is a flood of new medical graduates. Resources could be better allocated to training junior doctors in the provision of safe sedation in intensive care, radiology and emergency medicine.” “Before this model is adopted, research needs to show that an anesthesia-led nurse sedation model is equivalent or superior to our current model of care.” “Strong hospital support in funding this training programme will be needed. This model will need to be led and championed by the department of anesthesia.” “I do not possess the necessary skill set to train nurses in this role.” “There remains the issue of who is responsible for the ‘prescription’ of the medication’ This creates further logistically and training considerations.” “It will completely depend upon the suitability of the nurses chosen for the role.” “For this model to be safe, nurses need to train to the same level as Certified Registered Nurse Anesthetists (CRNAs) the USA. Anyone can give an anesthetic when things go well.” “This model should be applied only with clear and strict guidelines.”

Participants discussed the need for robust governance to ensure the safety of the proposed model. The complexity of patients and the potential need for emergency management were highlighted as significant concerns. The main themes and concerns identified with an anesthesia-led NP program were uncertainty regarding accountability and responsibility. In addition, there were strong concerns about whether professional indemnity insurance would be provided to anesthetists who were elected to cover sedation practices delegated to nursing staff, and whether anesthetists should “take ultimate responsibility for prescribing practices” even if acting in a supervisory role.

### Face-to-face interviews

From the face-to-face analysis, four major themes were identified from the data. These were complexity of patients or emergency management, economic impact, training/skill level of nurses, and, finally, other suggestions.

#### Complexity of patients/emergency management

The management of unforeseen deterioration was a common theme expressed by all participants who were interviewed. Although participants acknowledged the relative rarity of major adverse events in colonoscopy, there was a strong opinion that only anesthetists could expertly manage significant patient deterioration during the procedure. One respondent noted that there have been several endoscopies “near misses,” including aspiration, and severe laryngospasm in “healthy American Society of Anesthesiologists (ASA) class I–II patients,” which were the same patient characteristics suggested for this proposed anesthesia-led NP model. Most respondents thought that it would be very difficult to completely mitigate such risks, and even if strict criteria for an anesthesia-led ANP program were applied, complications could occur in even the healthiest of patients.

#### Economic impact

There appeared to be a strong perception that the motivation for the program was partly driven by costs. The respondents highlighted several economic considerations. The most common theme that emerged was that patient safety should not be traded for financial savings. One participant stated, “what is the cost of death if this is being done for costs savings?” Multiple respondents noted that there is a potential cost of reduced efficiency while training NPs in anesthesia, which might be avoided by improving the efficiency of the existing lists. Another area of concern was the risk of litigation and the costs associated with it, as the model falls outside the remit of the ANZCA guidelines.

#### Training/skill level of nurses

Most respondents discussed the need for advanced skills, particularly in airway management and intravenous cannulation in regard to the proposed anesthesia-led NP program. A further concern was that nurses strictly adhere to protocols; however, endoscopy cannot be easily reflected in a straightforward protocol. Procedural sedation requires fine-tuning and constant adjustment in response to the patient’s level of sedation throughout the procedure. Participants further highlighted that candidate selection would be crucial to the successful implementation of the program.

#### Alternative suggestions

A number of respondents suggested alternatives to the proposed model, such as a similar training program and a model of supervision for anesthetic trainees rather than nurses. One respondent suggested that they “use junior anesthesia trainees with 1:2 supervision.” Statements such as “why doesn’t the organisation employ more anesthetists to run additional lists?” were a common theme expressed by almost all participants. Their responses revealed a belief that nurse-led sedation would dilute training opportunities for anesthesia, emergency, and critical care residents and registrars, who also require training in sedation. Responses indicated a higher level of acceptability where training programs involved the training and supervision of anesthetic trainees. Indeed, the respondents suggested that a 1:2 supervision model with anesthetic trainees would contribute to improving the availability of anesthetic resources.

## Discussion

### Key findings

We performed a prospective, mixed methods survey of practicing fellow anesthetists in a university teaching hospital to examine their attitudes towards a proposed anesthesia-led NP program for low-risk patients undergoing colonoscopy. Despite the theoretical benefits of improved patient access to colonoscopy services, and in contrast to our hypothesis, we found that most anesthetists were not willing to participate in the supervision and training of nurse practitioners. The predominant themes underlying their lack of support for the program were a perception that patient safety would be compromised compared to the current model of anesthesia-led care, the model does not meet the ANZCA guidelines [22] for procedural sedation, and analgesia and the program may be a public liability prone to litigation in the event of an adverse outcome. Concerns about consumer acceptance and cost-effectiveness were raised, with numerous participants commenting that, before any program could be implemented, the model should be pilot tested to better understand consumer attitudes, logistical feasibility, patient and proceduralist attitudes, clinical governance, and, importantly, patient safety. Finally, we found that half of the participants acknowledged the current strain on resources and were in favor of exploring alternate solutions for colonoscopy efficiency.

### Relationship with previous studies

To date, no studies have examined the attitudes of anesthetists or other physicians towards nurse sedation models for colonoscopy. In an Australian context, three studies have examined the safety of a nurse-led sedation model for endoscopy. Ooi and Thomson [23] described “endoscopist-directed nurse-administered propofol sedation” for low-to-moderate-risk patients (ASA class < 3) undergoing endoscopic procedures at a tertiary referral center. Over 33 500 procedures were performed, including 17 146 colonoscopies, over a 9-year period. This model was reported to be cost-effective and safe. However, the authors provided little evidence to support their safety findings; the only metric for safety was whether the patient had a medical emergency team response following the procedure.

Recently, Sathanthan et al. [24] reported on the safety of physician-directed nurse-administered propofol sedation in low-risk patients undergoing endoscopy and colonoscopy. This prospective study was conducted at a single-center Australian teaching hospital and included 981 patients with an ASA class of 1–3 undergoing an endoscopic procedure. There were no reported major intra-procedural adverse events; however, 6.4% of patients experienced minor adverse cardiorespiratory (hypotension, hypoxia, bradycardia, or apnea) problems. In contrast to the perceived attitudes about safety concerns from participants in our study, Sathanthan et al. concluded that nurse-led procedural sedation is safe when patient selection is stringent and that the nurse training program is appropriate.

Finally, Jones et al. [25] described the initial efforts to establish a physician-directed nurse sedation program in two public Australian hospitals. The program had demonstrated a high level of safety, with no adverse incidents related to nurse sedation. However, reports of the safety of this program were anecdotal and not supported by clear evidence; the study focused on the satisfaction of key stakeholders involved in the program. Similar to our findings, patient safety was reported to be at the forefront of the non-anesthetists providing sedation. Indeed, this issue directly mirrors the area of concern that participants voiced in our study.

### Study implications

Our study implies that anesthetists are concerned about the safety, feasibility, and governance of a potential anesthesia-led NP model in an Australian teaching hospital. Our findings suggest that there is a strong sense of anesthetist “identity” in that the vast majority of participants who viewed their role as “specialist medical role expert in airway management” requiring complex skills and structured training, which made the specialty safe. These findings are reiterated by ANZCA, who state that patient safety should always be at the forefront and that anesthesia is a specialist medical role that requires complex skills and training in order to ensure the highest level of safety.

Due to the fact that propofol has serious and potentially fatal side effects that commonly include loss of airway reflexes and hemodynamic instability, out of concerns for patient safety, in 2012, the European Society of Anesthesiology formally retracted their endorsement of non-anesthetist administering propofol for gastrointestinal endoscopy [26]. Further, for the same reasons, 21 national anesthesia societies in Europe published a consensus statement confirming that propofol should only be administered by those trained in the administration of general anesthesia [27]. More recently, however, the European Society of Gastrointestinal Endoscopy, European Society of Gastroenterology, and Endoscopy Nurses and Associates updated their guidelines on the administration of propofol by non-anesthetists for patients undergoing gastrointestinal endoscopy and recommended that there should be primary involvement of an anesthetist for endoscopy procedures in the following patient circumstances: (i) ASA class ≥  3, (ii) Mallampati score ≥  3, (iii) any risk of airway obstruction, (iv) chronic opioid use, and (v) cases where prolonged endoscopy is anticipated [28].

Our findings further suggest that any economic impact of an anesthesia-led NP program is not likely to sway the anesthetist cohort; participants firmly relayed that the cost of any patient’s life supersedes any action to balance a budget, a sentiment mirrored in an ANZCA 2014 press release [29]. Finally, our findings imply that while most anesthetists are not willing to be involved in the supervision and training of nurse practitioners in sedation, most were in favor of exploring alternate solutions for colonoscopy efficiency. Proposed solutions included developing a model of care where one anesthetist supervises two anesthesia registrars/residents and training other junior doctors to be skilled in the provision of safe sedation, given that these skills are also relevant to other specialties, such as intensive care, hematology, radiology, and emergency medicine. Such a model would also be aligned with the ANZCA guidelines on sedation for diagnostic and interventional procedures [21].

### Strengths and limitations

Our study has several strengths. The proposal was developed in consultation with a multidisciplinary team with all key stakeholders, including gastroenterology, surgery, anesthesia, and nursing being supportive of the project. The working group met fortnightly over a 4-month period, allowing for transparent discussions of the logistical, pragmatic, and resource challenges of such a program. Further, hospital, departmental, and individual (nurse, patient, physician) determinants acting as barriers and enablers that could influence the implementation of such a program were identified. In turn, this allowed for a collaborative, hospital-wide, feasible framework for any potential change of current practice.

This is the first study to date exploring the attitudes of anesthetists working in a university teaching hospital towards an anesthesia-led NP model. The confidential, anonymous, and de-identified design of the survey may have encouraged respondents to be more willing to share personal information about their attitudes towards this proposed model of care. A high proportion of the anesthetist cohort completed the survey; therefore, our sample is likely representative of the broader consultant anesthesia group in our department. The use of free-text responses and face-to-face interviews allowed for more detailed qualitative analyses of some of the perceived benefits and barriers of the program.

However, we acknowledge that our study has some limitations. Anesthetists who were interested in the topic may have been more likely to participate, resulting in selection bias. It is possible that participants chose more peer-group desirable answers, which resulted in response bias. Importantly, the study was conducted at a single tertiary center, limiting the external validity of our findings to anesthetists at other hospitals. The involvement of other anesthetists and key external stakeholders (e.g., government agencies, other healthcare systems, insurance companies, and legislatures) in the working group stage may have assisted in the planning and the redesigning of anesthesia workflows and workforce roles relevant to this proposed model, which in turn may have encouraged different multidisciplinary teams to help change some of the anesthetist attitudes we observed. This may have also helped transform the attitudes and beliefs of anesthetists towards the proposed anesthesia-led NP model. Connecting other anesthetists at an early stage in the implementation process, with the aim of providing comprehensive, coordinated, continuous care, with improved quality and access for patient undergoing endoscopic procedures, and lower cost to the health care system, may have resulted in a different outcome. This was not undertaken, and we acknowledge this as a limitation of our study.

As the survey only included anesthetists, our findings are not generalizable to gastroenterologists or other proceduralists, surgeons, and nurses, and further research exploring the attitudes of these craft groups towards an anesthesia-led NP model should be explored in separate studies. As the anesthetists who participated in the survey and interviews are all working at the same hospital, their responses may be influenced by a cultural institutional bias. Finally, the face-to-face interviews were conducted by a neutral Austin employee, and participant responses may have, therefore, been aligned with the values set forth by the hospital.

## Conclusions

Most anesthetists working in a single-center university hospital did not support the proposed model of an anesthesia-led NP for patients undergoing colonoscopy. Patient safety, violations of the current ANZCA guidelines on procedural sedation, and logistical feasibility were significant barriers to the acceptance of the proposed model. Support for the ongoing exploration of alternate sedation models within the framework of the endorsed ANZCA guidelines for safe procedural sedation is common. However, the development of an anesthesia-led NP program would require careful negotiation with ANZCA, and future studies could focus on nurses, patients, and the proceduralist attitudes towards an anesthesia-led NP model.

## Data Availability

The datasets used and/or analyzed during the current study are available from the corresponding author on reasonable request.

## Abbreviations

NP: Nurse practitioner
ANZCA: Australian New Zealand college of anesthetists
ASA: American society of anesthesiologists
